# Current status of acupuncture and moxibustion in China

**DOI:** 10.1186/s13020-015-0041-1

**Published:** 2015-05-21

**Authors:** Min Yee Lim, Jian Huang, Baixiao Zhao, Lue Ha

**Affiliations:** School of Acupuncture-Moxibustion and Tuina, Beijing University of Chinese Medicine, Chaoyang, Beijing, 100029 China

## Abstract

Acupuncture and moxibustion are more integrated in the Chinese healthcare system than in the national healthcare systems of other countries. Development of acupuncture and moxibustion in China is making progress in this field. For overseas researchers, this commentary offers perspectives on the current status of acupuncture and moxibustion in China and examines relevant opportunities and challenges in healthcare reforms. There has been a steady increase in the number of undergraduates and postgraduates studying acupuncture and moxibustion in Chinese Medicine (CM) universities in China over the past decade. The legislation of CM physicians that was established in 1999 and the launch of continuing medical education in CM in 2002 have ensured the basic competency of practitioners. The Chinese Government has also shown support for CM development by increasing investment in related fields of research and administration. New challenges have emerged as the healthcare landscape in China has evolved over the past decade. It is important to harness the potential of acupuncture and moxibustion to create a value-driven healthcare system that meets the health needs of a rapidly aging society.

## Background

The Chinese term *zhēn jiū* refers to acupuncture (*zhēn*) and moxibustion (*jiū*), although acupuncture is the more widely recognized treatment among the plurality of acupuncture-related treatments that include moxibustion, cupping, bloodletting, and scraping. Acupuncture and moxibustion are based on the theory of channels and collaterals in Chinese Medicine (CM). Their effects are exerted by the stimulation of acupuncture points at specific anatomic locations on the body surface, or tender points known as *ashi* points, usually by needles or ignited moxa floss. Interest in acupuncture and moxibustion waned in China from the seventeenth century, and their practice was not included in the Imperial Medical Institute by decree of the Emperor in 1822 [1]. However, since the establishment of the People’s Republic of China in 1949, the Chinese Government has supported the development of acupuncture and moxibustion. In the 1970s there was excitement over acupuncture analgesia and the discovery of the release of neurotransmitters during acupuncture [2]. Acupuncture and moxibustion are now well integrated into the Chinese healthcare system and are practiced along with conventional Western medicine in China [3].

Development of acupuncture and moxibustion in China will influence the overall dynamics and progress of this field. We would like to comment on the current situation of acupuncture and moxibustion in China and examine their opportunities and challenges for healthcare reform.

## Healthcare system related to acupuncture and moxibustion in China

The Chinese health prevention and delivery system is based on a three-tier system developed in the 1950s that includes hospitals, health centers, and clinics [3]. Since 1999, the government has promoted the development of Community Health Services Centers as major providers of primary healthcare, which includes the practice of acupuncture and moxibustion. There are currently 4169 CM hospitals in China (including integrated CM and Western medicine hospitals), and more than 90 % of these hospitals have an acupuncture and moxibustion department [4].

The three main health insurance programs in China are the Urban Employees Basic Medical Insurance (UEBMI) initiated in 1998, the Urban Resident Basic Medical Insurance (URBMI) for urban residents without formal employment, and the New Cooperative Medical Scheme (NCMS) for rural residents [5]. These insurance programs cover specific groups and have different funding and operation regulations; thus, there are varying levels of financing and reimbursement ratios for acupuncture and moxibustion treatments in China [6].

## Acupuncture and moxibustion professionals in China

### Education

Higher education in acupuncture and moxibustion is offered by CM, Western medical, and non-medical universities in China. There are presently 46 tertiary CM universities (the majority of which have acupuncture and moxibustion programs), plus 10 Western medical and six non-medical universities offering acupuncture and moxibustion programs [4]. The tertiary education program for acupuncture and moxibustion has matured into a system offering courses of different lengths for Associate, Bachelor's, Master's, and PhD degrees to meet the health needs of the country. The most common education route is a stepwise system in which students spend 5 years studying for a Bachelor's degree plus 3 years each for Master's and PhD degrees, or complete a 7-year Master's program (combining undergraduate and Master's courses).

There has been a steady increase in the number of undergraduates studying acupuncture and moxibustion in CM, Western medical, and non-medical universities from 2002 to 2012 in China [7]. There were 30,496, 7533 and 2899 undergraduates enrolled in CM, Western medical, and non-medical universities, respectively, in 2012, compared with 10,586, 1180 and 526, respectively, in 2002 [7]. The enrollment of postgraduates in acupuncture and moxibustion in CM universities has also shown an increasing trend over the past decade, from 696 to 2171 postgraduates [7]. In contrast, the postgraduate intake for Western medical universities showed a small increase from 14 to 80, while the intake for the non-medical universities decreased from 29 to 4 over the past decade.

### Licensing

The legislation of CM physicians was established in 1999 to outlaw unqualified and unreasonable practice, and is now under the management of the State Administration of Traditional Chinese Medicine (SATCM) [8]. A candidate for CM practitioner qualification must possess a Bachelor's (or higher) medical degree from a tertiary institution and complete at least 1 year of probation under supervision of a registered practitioner in a healthcare institution to qualify for the physician’s licensure examination. Assistant physicians may qualify for the physician’s licensure examination if they possess an assistant physician practicing certificate plus an Associate medical degree from a tertiary institution; they must also have 2 years of relevant experience in a healthcare institution, or 5 years of relevant experience for candidates from vocational institutions [8]. Additionally, those without professional medical qualifications who have apprenticed for 3 years under a mentor with over 15 years of CM clinical experience can qualify for the Apprenticeship Assessment, while those with recognized expertise or outstanding skills who have been engaged in lawful clinical practice of CM for more than 5 years can qualify for the Recognized Competence Assessment to obtain their physician’s practicing license [9]. The Apprenticeship Assessment is organized by relevant CM organizations at the provincial level, while the Recognized Competence Assessment is managed at the municipal level. The physician’s licensure examination contains two components: theoretical and practical. The practical component requires candidates to demonstrate basic proficiency in acupuncture and moxibustion, including needling, moxibustion, cupping, and scalp and auricular acupuncture [10]. The theoretical component is a 600 multiple choice-based examination administered over a 2-day period that assesses the knowledge of the candidate in four categories, each containing 150 questions [11]. The first category covers the classics of CM, basics and diagnostics of CM, Chinese materia medica, and formulary. The second category covers basic diagnostics, internal medicine and epidemiology of Western medicine, and medical ethics and legislation, to ensure competence of candidates in the understanding of clinical science and medical ethics considered essential for provision of healthcare. The third and fourth categories cover CM-related clinical topics that include CM internal and external medicine, gynecology, pediatrics, and acupuncture and moxibustion.

Continuing medical education (CME) was established in 2002 under the management of the SATCM and is coordinated by CM institutions to improve the competency of practitioners and ensure the passing down of knowledge. Registered physicians are required to earn at least 25 CME credits each year through mentorship learning, distance learning, registered meetings or courses, or publication of medical literature. Mentorship learning aims to pass CM clinical experience from senior to junior physicians.

## Acupuncture and moxibustion funding development in China

Under the supervision of the Chinese Ministry of Science and Technology, the National Key Technology Research and Development Program launched in 1982, the 863 Program in 1986, and the National Basic Research Program (also called the 973 Program) in 1997 are major sources of funding for acupuncture and moxibustion research. At the end of the “11^th^ five-year plan” (2006–2010), the National Key Technology Research and Development Program funded two acupuncture and moxibustion clinical trials at a cost of 113 million CNY, plus 18 projects related to standardized manipulations of acupuncture and moxibustion [4]. The 973 Program funded six projects related to basic research into acupuncture and moxibustion at a cost of 108 million CNY [4].

The National Natural Science Foundation of China (NSFC) is an organization directly affiliated with the State Council that aims to fund and encourage basic and applied research in China. In 2013, the NSFC funded 109 acupuncture and moxibustion projects at a cost of 57 million CNY [12]. The majority of these projects were predominantly for scientific research into the mechanism of action of acupuncture and moxibustion. The remaining projects covered investigations into the nature of channels and collaterals, acupuncture points, acupuncture analgesia, and the efficacy of different needling and moxibustion practices for different clinical conditions.

## Opportunities and challenges

The State No. 22 Documents related to TCM development was enacted in 2009 with the aim to promote the TCM industry in China [13]. New challenges have emerged as the healthcare landscape in China has evolved over the past decade.

### Characteristics of acupuncture and moxibustion practice

China has been experiencing a marked increase in demand for healthcare, complicated by surging healthcare costs and an aging population. The use of acupuncture and moxibustion can help to contain healthcare costs, as these treatments are usually less expensive and more accessible than other treatments for disease prevention, health promotion, and chronic non-communicable diseases [14]. In 2011, a survey reported that the average acupuncture treatment fee was 19.32 CNY [15]. However, a balance has to be achieved between providing affordable care and developing the sector in the country. The same survey reported that the majority of acupuncture and moxibustion practitioners earn an average of 1000–1999 CNY [15]. Low pay, long working hours, and excessive workload are some of the longstanding problems faced by acupuncture and moxibustion practitioners in China [16]. Another problem is that the relationship between patients and physicians has greatly deteriorated because of the vicious cycle that has ensued from the commodification of the healthcare system [17]. These issues pose serious implications, as there are inadequate incentives to attract and retain practitioners.

### Acupuncture and moxibustion education

The provision of a university education should have a strong nexus with the needs of the healthcare industry to enhance the capabilities of graduates. The medical education system underwent rapid expansion to meet the health needs of the country, as evidenced by the three-fold increase in yearly enrollment of acupuncture and moxibustion undergraduates in CM universities over the past decade [7]. In recent years, different specialized fields of acupuncture and moxibustion study at the undergraduate level, such as in cosmetology and rehabilitation, have also been offered [7]. The medical education strategy aspires to produce a health workforce comprising clinical staff for rural services, primary care clinicians, and high-quality academic researchers and practitioners by providing medical degrees of different lengths [18]. As a specialized field of study, the undergraduate curriculum focuses solely on the content of the discipline to ensure a basic level of core competency among students. However, the changing medical landscape presents different healthcare challenges compared with those faced in the past, and the new generation of acupuncture and moxibustion practitioners are equipped with an understanding of modern medical knowledge such as physiology and neurology. Additionally, there is growing consensus in all healthcare systems that evidence-based practice is the most responsible course of action for improving health outcomes [19]. Evidence-based medicine requires the knowledge of the exact chemistry of the drug used, the physical and chemical activities involved, and most importantly, the biological responses of the recipient [20]. The channels and collaterals might be a functional rather than an anatomical concept that involves a summation of multiple physiological functions including the nervous, circulatory, endocrine, and immune systems [21]. Therefore, it is important that the training curriculum recognizes the need for the inclusion of modern medical training as well as traditional content. Moreover, excellence in university education has been linked with interdisciplinary learning. Besides producing qualified practitioners, medical training should give more consideration to the soft skills and humanities that are often neglected in the curriculum and should allow potential practitioners to appreciate insights from other fields. CM education is now seeing a need to combine seeming opposites: the introduction of new themes into the education program such as modern science and soft skills training while not neglecting traditional topics, and an emphasis on core competency while establishing a scientific basis for the research and practice of acupuncture and moxibustion.

### Acupuncture and moxibustion internationalization

The internationalization of acupuncture and moxibustion presents new frontiers for the field and higher education institutions. Among the different CM treatment modalities, acupuncture and moxibustion are the most used and recognized worldwide [22]. Problems such as poor implementation and applicability of the standards, talent deficiency, and lack of relevant basic research have impeded the progress of international standardization of acupuncture and moxibustion [23]. Nonetheless, the recent publication of the International Organization for Standardization (ISO) standard for sterile acupuncture needles for single-use marked the international recognition of the acupuncture needle, which has been in use for thousands of years [24]. In terms of acupuncture and moxibustion education, many CM universities have introduced acupuncture and moxibustion programs for international students. However, the diversity of these students ranges from trained Western medical doctors to those with no prior medical training, including those who are older and seeking a career change. Some also look to China to seek certification for short-term advanced studies and introductory courses. Besides structuring training content to accommodate the different student groups, the pedagogy also has to be tailored to the learning styles of foreigners. Regulations also have to be in place for administrative issues such as enrollment requisites, curriculum, and accreditation to maintain the standard of acupuncture and moxibustion courses offered by Chinese universities.

### Acupuncture and moxibustion research

Cultural and social influences have affected the research trends and priorities in China compared with the West. Much of the research related to complementary and alternative medicine in the West aims to establish scientific evidence regarding the efficacy of acupuncture and moxibustion by using sham or placebo controls. Such research aims to establish the evidence base guiding the provision of acupuncture-related treatment modalities in clinical practice. However, the lack of convincing evidence from rigorous research is a major stumbling block preventing the widespread acceptance of acupuncture and moxibustion. On the contrary, acupuncture and moxibustion are well accepted in China and hence research priorities are strongly associated with clinical practice and more geared toward understanding the underlying mechanisms. Practitioners are also keen on improving the effectiveness of their practice, leading to the development of new techniques such as balanced acupuncture, abdominal acupuncture, and acupotomy, and the development of new equipment such as electroacupuncture stimulators and infra-red moxibustion devices [25–27]. However, any form of treatment, especially novel techniques based on modern theories, needs to go through scientific scrutiny before it is considered acceptable. For this reason, randomized controlled trials of abdominal acupuncture have been conducted to study the effectiveness of this newly developed technique [28–30]; without evidence-based clinical practice screening, a traditional treatment practice could only be taken as a form of alternative treatment [20]. While these developments reflect recent advances in the field of acupuncture and moxibustion, differences in practice, training, and research priorities between China and the West may present challenges to international research collaborations.

## Conclusions

The challenges ahead are large, as the factors involved are intertwined with no easy solution. It is important to harness the potential of acupuncture and moxibustion to create a value-driven healthcare system that meets the health needs of a rapidly aging society. The policies related to acupuncture and moxibustion must also be aligned with China’s health reforms, and there must be the will to see it through for the reforms to be successful and sustainable.

## Abbreviations

CM: Chinese Medicine
CME: Continuing medical education
ISO: International Organization for Standardization
NSFC: National Natural Science Foundation of China
NCMS: New Cooperative Medical Scheme
SATCM: State Administration of Traditional Chinese Medicine
UE-BMI: Urban Employees Basic Medical Insurance
URBMI: Urban Resident Basic Medical Insurance
